# Intraspecific variation in metabolic responses to diverse environmental conditions in the Malagasy bat *Triaenops menamena*

**DOI:** 10.1007/s00360-025-01608-1

**Published:** 2025-03-20

**Authors:** Sina Remmers, K. Dausmann, M. Schoroth, H. Rabarison, S. Reher

**Affiliations:** Functional Ecology, Institute of Cell and Systems Biology of Animals, Universität Hamburg, Hamburg, Germany

**Keywords:** Bats, Torpor, Intraspecific variation, Physiological flexibility, Madagascar, Roost comparison

## Abstract

Widespread species often display traits of generalists, yet local adaptations may limit their ability to cope with diverse environmental conditions. With climate change being a pressing issue, distinguishing between the general ecological and physiological capacities of a species and those of individual populations is vital for assessing the capability to adapt rapidly to changing habitats. Despite its importance, physiological variation across broad range distributions, particularly among free-ranging bats in natural environments, has rarely been assessed. Studies focusing on physiological variation among different populations across seasons are even more limited. We investigated physiological variation in the Malagasy Trident Bat *Triaenops menamena* across three different roost types in Madagascar during the wet and dry season, examining aspects such as energy regimes, body temperature, and roost microclimates. We focused on patterns of torpor in relation to roosting conditions. We hypothesized that torpor occurrence would be higher during the colder, more demanding dry season. We predicted that populations roosting in more variable microclimates would expend less energy than those in mores stable ones due to more frequent use of torpor and greater metabolic rate reductions. Our findings highlight complex thermoregulatory strategies, with varying torpor expression across seasons and roosts. We observed an overall higher energy expenditure during the wet season but also greater energy savings during torpor in that season, regardless of roost type. We found that reductions in metabolic rate were positively correlated with greater fluctuations in ambient conditions, demonstrating these bats’ adaptability to dynamic environments. Notably, we observed diverse torpor patterns, indicating the species’ ability to use prolonged torpor under extreme conditions. This individual-level variation is crucial for adaptation to changing environmental conditions. Moreover, the flexibility in body temperature during torpor suggests caution in relying solely on it as an indicator for torpor use. Our study emphasizes the necessity to investigate thermoregulatory responses across different populations in their respective habitats to fully understand a species’ adaptive potential.

**Supplementary Information:**

The online version contains supplementary material available at 10.1007/s00360-025-01608-1.

## Introduction

Most animals are restricted to environments with specific characteristics, which determine their overall distribution, abundance and behaviour (Segal et al. 2021; Matthiopoulos 2003; Wilmé et al. 2006). More widespread species often experience a variety of environmental conditions across their range and thus, are often considered to be generalists. However, some populations may be locally adapted and therefore unable to cope with the whole spectrum of characteristics present across the species range (Reher et al. 2022a; Vences et al. 2009).

Seasonal fluctuations in abiotic conditions and resources within a habitat can greatly impact energy expenditure and behavior (Bryce et al. 2022; Heldmaier et al. 2013). Yet, maintaining a balanced energy budgets is crucial for survival, suggesting that physiological specialization of populations to distinct habitat conditions may provide benefits when facing energetic challenges, counteracting the need for seasonal migration or other behavioral strategies (Bartolini and Giomi 2021; Nowack et al. 2023; Wong and Candolin 2015). However, specific adaptations to microclimates and habitats could hinder the ability to adjust to rapidly changing environments, which often necessitates a broader physiological scope (Reher et al. 2022a; Spence and Tingley 2020).

In endotherms, thermoregulation is one of the most energy consuming physiological processes. Various strategies have evolved to minimize this expenditure, with heterothermy being one of the most effective ones (Dausmann 2014; Heldmaier et al. 2013; Stawski et al. 2014). Minimized energy expenditure is achieved by entering torpor, a controlled and reversible state of hypometabolism that can vary in expression and duration, ranging from micro bouts (lasting around 20 min) and daily torpor (short torpor bouts with a diurnal cycle) (Reher et al. 2018), to prolonged torpor (up to several days) (Kobbe et al. 2011) or hibernation (several months) (Geiser 2004; Nowack et al. 2020; Stawski 2012). The data from the last decades have shown that heterothermy can be used advantageously in both cold and hot climates (Dausmann et al. 2005; Geiser 2021; Ruf and Geiser 2015).

Torpor can be induced by changes in ambient temperature that fall below or rise above the thermoneutral zone (*TNZ*). The *TNZ* is the temperature range in which endotherms do not have to spend additional energy to maintain their body temperature within the normal range that is ideal for its physiological and metabolic processes, known as euthermia (Geiser 2021; Hill et al. 2022). Recent studies indicate that torpor is employed to cope with various ecological or physiological bottlenecks, some of which are pertinent in the context of climate change (Nowack et al. 2017). Sugar gliders (*Petaurus breviceps*) use torpor during storms when unable to forage (Nowack et al. 2015) and grey mouse lemurs *Microcebus murinus* use torpor to conserve water (Schmid and Speakman 2009). However, there are also disadvantages and risks associated with the use of torpor, such as oxidative damage during arousals (Staples and Brown 2008), increased predation risk if not concealed (Radzicki and Hejduk 1999), missed social opportunities (Choi et al. 1998) or generally diminished responsiveness to environmental stimuli (Geiser et al. 2018) among others (Bouma et al. 2010; Giroud et al. 2021).

Many small endotherms, bats in particular, are known to employ torpor throughout the year, and thus experience a wide range of body temperatures both seasonally and daily while thermoconforming to ambient temperature during torpor (Dunbar and Brigham 2010; Levesque et al. 2016). Indeed, the largest range in skin temperature by a mammal has been documented for the Australian desert bat *Mormopterus petersi*, varying between 45.8 °C during a heat wave and as low as 3.3 °C during the winter season (Bondarenco et al. 2014). Although torpor can occur throughout the year, its propensity often increases during colder and/or drier seasons, when food availability is reduced. This is especially true for insectivorous species, as demonstrated by studies on the subtropical eastern long-eared bat *Nyctophilus bifax* (Stawski and Geiser 2010b).

Bats are often widespread and their roosting types and prevailing microclimatic conditions vary locally. To cope with different environmental requirements, they exhibit intraspecific physiological variation and flexibility relative to the specific conditions of their habitat (Dunbar and Brigham 2010; McGuire et al. 2022; Reher et al. 2022a). It is well established that roost conditions are especially important for bats as shelters from inclement weather and predators (Furey and Racey 2016), mating places (Page and Dechmann 2022) and maternity roosts (Sedgeley 2001). Even though species specific roost preferences regarding micro-climatic conditions exist for many bats, multi-species assemblages are also common, especially in tropical cave systems (Cardiff 2006; Rodriguez-Duran 1998). Large colonies can be beneficial for thermoregulation (e.g. huddling decreases heat loss and EWL) and reducing predation risk (Czenze et al. 2017a; Furey and Racey 2016).

The preference for a particular roost type and physiological patterns may vary locally among populations, but also within individuals of a population over the year. The lesser short-tailed bat *Mystacina tuberculata* in New Zealand for instance, changes roost sites seasonally, preferring communal roosts during summer and solitary roosts during winter. Moreover, it uses torpor flexibly, varying from prolonged torpor bouts (up to 120 h) in winter to shorter daily torpor bouts in summer (Czenze et al. 2017a) and separate populations of *M. tuberculata* exposed to different environmental conditions use different energy saving strategies, including the way torpor is expressed (Czenze et al. 2017b).

Similarly, differences in torpor patterns and physiological variation within different populations of the Malagasy bat species *Macronycteris commersoni* align with habitat and roost options and preferences (Reher et al. 2022). This species inhabits the same habitats and even shares some roosts with our study species *Triaenops menamena*. The Malagasy Trident Bat *T. menamena* is endemic to Madagascar, but widely distributed along the whole western region of the island. Within this range, it lives in habitats with a variety of climatic conditions and roosting opportunities from evergreen rainforest to dry deciduous forest and dry spiny thickets (Goodman 2011). Therefore, *T. menamena* is a good model species for our research questions, because it is found in large numbers across diverse habitats with varying microclimates. Their distribution provides an opportunity to examine intraspecific variation in physiological responses. Furthermore, patterns and flexibility in thermoregulation and torpor use have already been demonstrated in the hibernating bat species *M. commersoni* (Reher et al. 2018). This makes it particularly interesting to investigate how non-hibernating species like *T. menamena* respond to different roost conditions and seasonal changes in microclimates, while being active year-round.

In the context of ongoing climate change, it is important to differentiate between the general ecological and physiological potential of a species as a whole and that of individuals or populations, to assess rapid adaptability to changing habitats within evolutionary short time frames. However, most studies do not account for physiological variation, species’ energy budgeting or thermoregulation across a broad distribution range (McGuire et al. 2022; Dunbar and Brigham 2010). This adaptive physiological potential is best studied within natural habitats, with all of the relevant constraints and possibilities. Yet, there are only few studies on heterothermy and hypometabolism in free-ranging bats in their natural environment (Liu and Karasov 2011; Reher et al. 2018; Stawski and Currie 2016; Turbill et al. 2003). Moreover, studies on different populations and across seasons are even more limited, restricting conclusions about physiological adaptations, as thermoregulatory responses from one site might not accurately represent the species as a whole.

We investigated physiological variation within different populations of *T. menamena* from three distinct roosts and across seasons, by comparing energy regimes (metabolic rate; $\:\dot{V}$*O*_*2*_), skin temperature (*T*_*sk*_) and roost microclimate. We focused on patterns of torpor in relation to roosting conditions and changes in ambient temperature, to examine thermoregulatory capacity and intraspecific physiological variability. We hypothesized, that the occurrence of torpor would be higher during the colder and typically more demanding dry season due to colder temperatures and lower food availability. We predicted that populations roosting in more variable microhabitats would generally expend less energy than those in more stable environments due to a higher frequency of torpor and greater reductions in metabolic rate, and that they would use different thermoregulatory responses and torpor patterns to maintain energy balance, demonstrating adaptive potential and intraspecific variation.

## Methods

### Study species

*Triaenops menamena* (Chiroptera: Rhinonycteridae) is a medium-sized species endemic to Madagascar (Goodman 2011). Females have a body mass (BM) ranging from 6.6 to 11.5 g, while males range from 8.2 to 15.5 g, with forearm lengths (FL) of 46–53 mm and 50–56 mm, respectively (Ranivo 2006; Peterson et al. 1995). This bat is common along the west coast, especially in the southern dry regions with spiny forests, but also inhabits western deciduous forest and the rainforest in the Northeast (Goodman et al. 2005). It typically roosts in caves, often forming large colonies of up to 41,000 individuals (Goodman 2011). However, when caves are not available, it will also use hollow trees in denser forests patches (Racey et al. 2009; Reher et al. 2019). *Triaenops menamena* primarily feeds on Lepidoptera, which can comprise up to 50% of its diet during austral winter, yet also consumes Coleoptera, Hymenoptera, Hemiptera and other insects if available (Rakotoarivelo et al. 2007; Bambini et al. 2010).

### Study area

This study was conducted in the drier regions of Madagascar in the south and the west, where rainfall is mainly confined to the warmer wet season (between 80 and 95% of the annual precipitation from October-March). However, rainfall is highly unpredictable, especially in the south, where it sometimes does not rain for several years (Rasoloariniaina et al. 2015). Food and water availability are greatly affected by the seasons and are much lower during the colder dry season (April-September) (Kobbe et al. 2011).

**Fig. 1:**
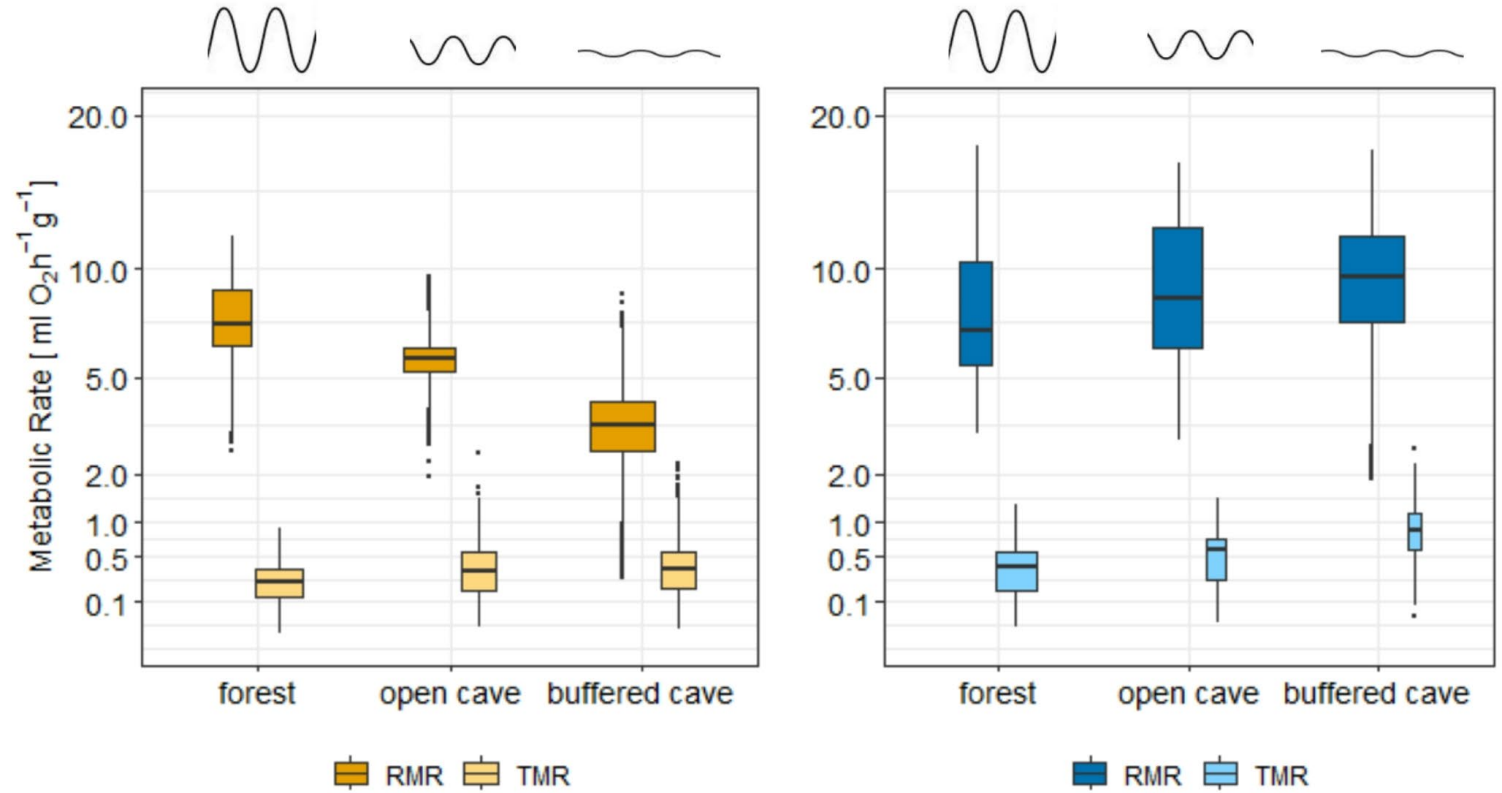
Resting metabolic rate (RMR) and torpid metabolic rate (TMR) for both seasons (yellow = dry season; blue = wet season) in the different roosts, plotted on a log-10-transformed Y-axis. The width of the boxplots is proportional to the sample size and amplitudes above the plots indicate the degree of environmental fluctuation

**Fig. 2:**
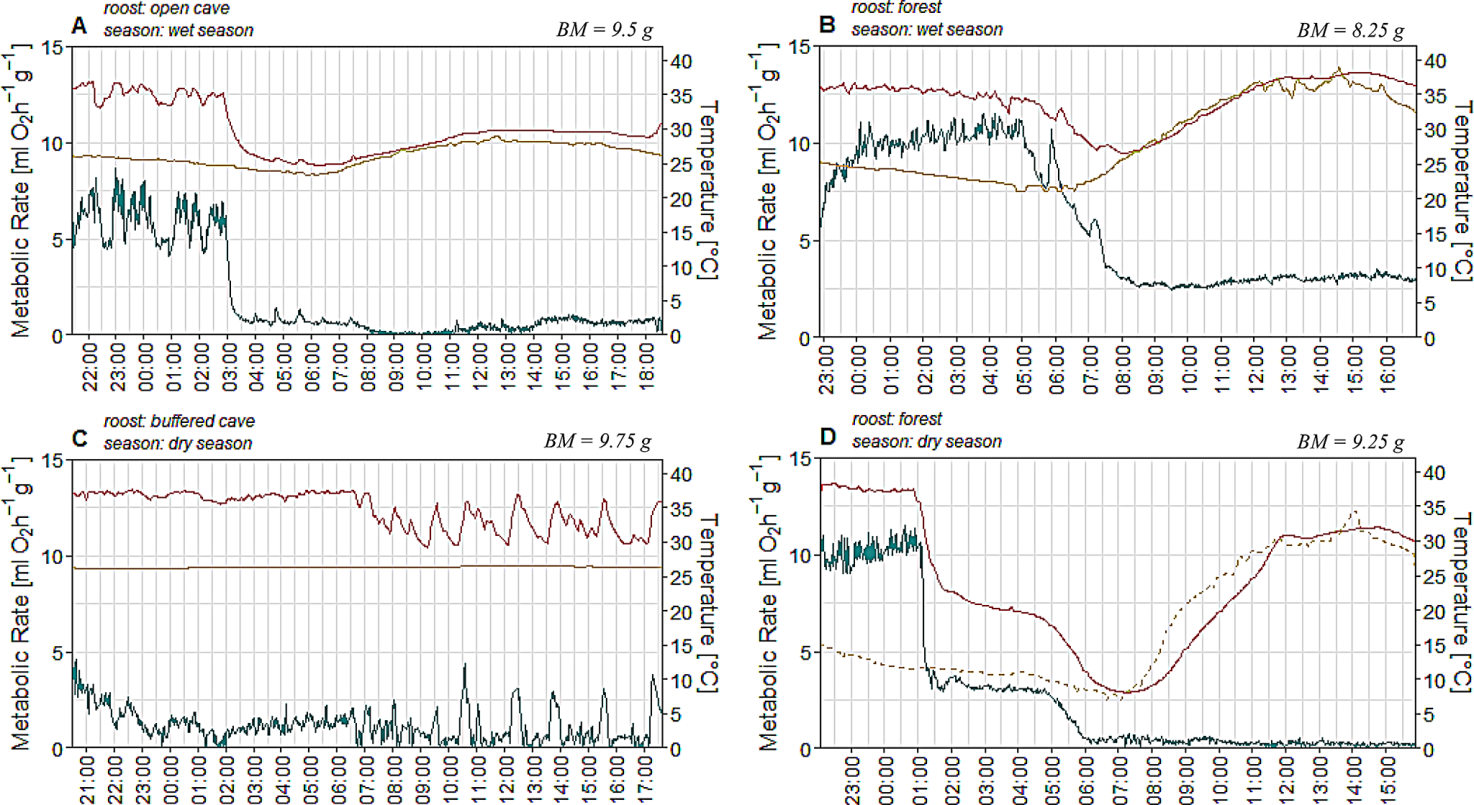
Metabolic rate , skin temperature  and ambient temperature  over the course of a day from four individuals demonstrating various torpor patterns, including (**A**) deep torpor, (**B**) shallow torpor, (**C**), short torpor bouts and (**D**) a combination of shallow & deep torpor expressions. The dashed line for ambient temperature in Fig. **D** illustrates measurement inaccuracies due to a misplacement of the logger. Absolute individual body masses are shown in the top right corner of each graph

We studied *T. menamena* populations using three different roosts along a gradient of microclimate variation (Supplements: Map). One study site was in the Kirindy / CNFEREF forest (20.06714°S / 44.65745°E) and included the roosts with the highest daily variation in environmental conditions; we refer to this roost as *forest*. The Kirindy forest is characterized by a dense, dry deciduous forest growing on sandy soil. In contrast to Tsimanampetsotse National Park, the Kirindy forest has no lime karst and therefore no cave systems. Thus, *T. menamena* likely roost in tree cavities and open vegetation. Unfortunately, we were unable to locate their exact roosts and therefore we lack information about their roosting behaviour in the forest, such as whether they form larger aggregations or prefer solitary roosts. The other study site was in the Tsimanampetsotse National Park, located on the Mahafaly plateau. This calcareous plateau is mainly covered in dense spiny forest and contains large numbers of caves and sinkholes, many of which contain water and underground steam systems (Rasoloariniaina et al. 2015). The two roosts used by the bats were located only 270 m apart but exhibit highly divergent environmental conditions and habitat features (Supplements Figs. 1 and 2). One roost is an open sinkhole with an adjoining cave, therefore more affected by ambient conditions. The other roost is a well-insulated cave, consisting of several connected chambers underground that are partially flooded, with constant high temperature and humidity. We refer to those roosts as *open cave* (24.04383°S / 43.75519°E) and *buffered cave* (24.04585°S / 43.75396°E), respectively. Even though these two roosts are in close proximity, we never recaptured individuals at a different roost from where they were initially captured during either the dry or wet season. However, we recaptured several individuals multiple times within the same area of the respective cave where they were initially captured. Therefore, we assume they do not switch roosts.

### Animal capture and handling

In the forest, bats were captured from February to March 2018 during the wet season and in July 2018 during the dry season using two to three mist nets (3 m high × 6 m long, 19 mm mesh) placed along transects. In the caves, bats were captured from February to March 2017 during the wet season and from June to July 2019 during the dry season using a two-bank 4 m^2^ harp trap. This trap was positioned at the cave entrance or main flight corridors. Traps and nets were set up about half an hour before sunset and left open for up to three hours, with continuous checks. To measure the bats’ metabolic rate while they are non-digestive, we ensured that we captured them as they were leaving their roosts for foraging, and we did not provide any food before starting respirometry. On each trapping night, two adult, non-reproductive individuals with a minimum body mass of 8 g had measurements of metabolic rate undertaken simultaneously (see below); individuals below the body mass threshold were released immediately upon capture. Individuals selected for respirometry were marked with a wing membrane tattoo, weighed (*BM*), sexed and forearm length (*FL*) was measured.

### Metabolic rate measurements / Respirometry

Mass-specific metabolic rate ($\:\text{i}\text{n}\:\stackrel{\prime }{V}$_*O2*_*in ml h*^*− 1*^*g*^*− 1*^) was measured using an open flow respirometry system. Bats were individually transferred into 2 l plastic metabolic chambers, which were darkened from the outside and provided with a net for the bats to hang from. All measurements were performed in the bats’ roosting environment, i.e. in the respective cave or forest. Metabolic rate was determined by measuring oxygen consumption using a portable differential oxygen analyser (OxBox; designed and constructed by T. Ruf and T. Paumann, University of Veterinary Medicine Vienna, Austria), powered by a car battery. The OxBox was connected to the metabolic chamber via gas-tight tubing (Tygon, Saint-Gobain COOP, Courbevoie, France.) and calibrated before and after each field season (*Supplements: Calibration*).

**Fig. 3:**
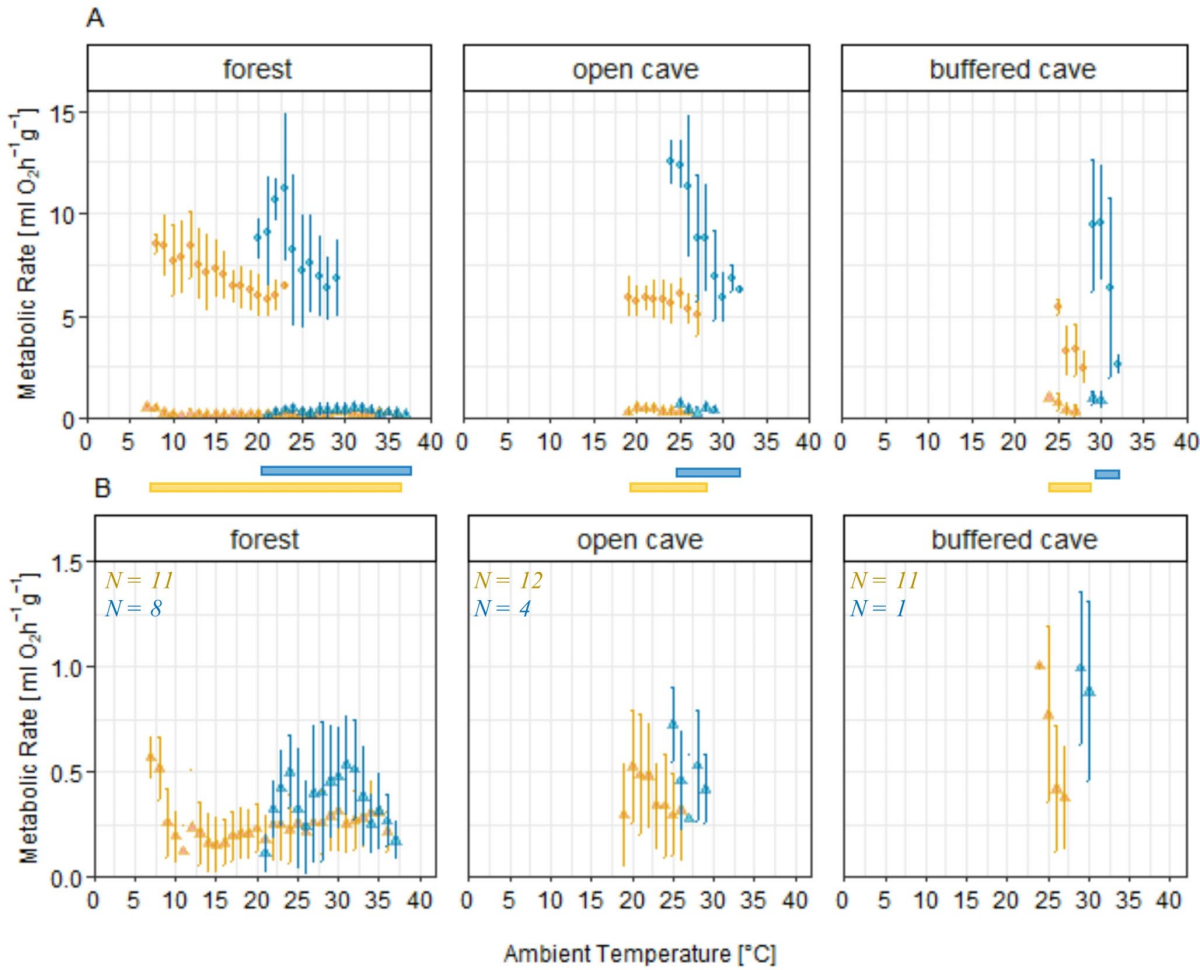
**A**: Average MR as function of T_a_ for the dry season (yellow) and the wet season (blue), shown separately for RMR (circles) and TMR (triangles). Average MR is presented as mean values for each 1 °C interval in T_a_, including error bars for respective standard deviation. **B**: Average TMR with standard deviation displayed on a finer Y-axis scale, including the number of torpid individuals (N). The bars between the graphs indicate the exhibited T_a_ range for each roost, separated for seasons

Respirometry was started immediately after capture and data were collected every 10 s for approximately 24 h. Each hour of respirometry consisted of 55 min of oxygen measurement inside the metabolic chamber (sample air) and 5 min outside next to the metabolic chamber (reference air). To evoke biologically relevant metabolic responses, respirometry was performed under the bats’ natural roost conditions of temperature and humidity inside their respective caves or in the forest. Therefore, the sample air and reference air were dried and filtered with silica gel before entering the gas analyser, but not before entering the metabolic chamber, with a constant flow rate of 50 l/h (Supplements: Fig. 3). The bats were then reweighed before being released at their capture sites during their usual active period.

### Temperature measurements

To measure *T*_*sk*_, a small patch of fur was removed between the shoulder blades and temperature-sensitive radio transmitters (0.45 g) were attached with a medical-grade latex adhesive (Osto-Bond, Montreal, Canada or Manfred Sauer GmbH, Lobbach, Germany; *Supplements: Calibration*). A remote data logger (DataSika SRX-800-D, Biotrack, Wareham, UK) was used to record *T*_*sk*_ every minute during respirometry. *T*_*sk*_ is an adequate proxy for body temperature in small-bodied animals, especially during resting phases (Audet and Thomas 1996; Dausmann 2005; Willis and Brigham 2003).

To determine environmental conditions, loggers (DS1923-F5# Hygrochron iButtons, Maxim integrated, San Jose, USA) were placed inside the metabolic chambers to record temperature (*T*_*a*_) and relative humidity (*RH*_*a*_) every 5 min during each measurement run. Additionally, long-term ambient temperature and *RH* were recorded continuously once an hour at each roost type in the wet as well as the dry seasons (Supplements Figs. 1 and 2).

### Data analyses

The OxBox output values were corrected for drift using the program Clampfit v10.7.0.3 (Molecular Devices, Sunnyvale, USA), with the hourly reference air measurements serving as a baseline, and further transformed with a box-specific internal adjustment value. The oxygen consumption ($\:\stackrel{\prime }{V}$_*O2*_) was corrected to standard temperature and pressure and calculated as ml O_2_ per hour following Eq. 11.2 in (Lighton 2008):

$$\:{\dot{V}}_{O2}=\frac{{FR}_{e}\:{(F}_{i}{O}_{2}-{{F}^{{\prime\:}}}_{e}{O}_{2})}{\left(1-{F}_{i}{O}_{2}\right)\:1-RQ}$$

with FR_e_ as overall excurrent flow rate and F_i_O_2_ as the fractional concentration of incurrent oxygen (for ambient air: F_i_O_2_= 0.2095). This equation is already considering the scrubbing of water vapor with silica gel juts before the oxygen analyzer (here: F_i_O_2_– F’_e_O_2_ = ∆volO_2_/100). The respiratory quotient (RQ) is the ratio of CO_2_ emitted to O_2_ consumed, which compensates for CO_2_ diluting O_2_ if it is not removed from the gas before entering the oxygen analyser (here: RQ = 0.85; reflecting a metabolic combustion of 50% fat and 50% carbohydrate) (Lighton 2008; Dausmann et al. 2009). Individual body mass (BM) was factored into the conversion to mass-specific metabolic rate (*MR*) in $\:\stackrel{\prime }{V}$*O*_2_ ml h^− 1^g^− 1^.

Body temperature thresholds are often used to define torpor bouts (Boyles et al. 2011; Brigham et al. 2011; Willis 2007). However, as there are variety of torpor patterns in tropical bats (e.g. “hot” torpor with high *T*_*sk*_) and *MR* patterns can vary even within populations, it was not possible to derive and apply a *T*_*sk*_ or *MR* threshold for torpor bouts in our data set. We therefore had to manually categorize torpor bouts by looking for rapid reductions in *MR* followed by periods of low values with very small amplitudes of *MR* fluctuations. When *T*_*sk*_ followed the same pattern as *MR* or thermoconformed to *T*_*a*_ during those identified torpor bouts and was not maintained at the stable level of euthermic *T*_*sk*_ of around 35 °C, it supported the classification of torpor, as thermoregulation is altered during torpor (Ambler et al. 2022; Levesque et al. 2016). Furthermore, we defined a *MR* reduction during torpor of over 70% of the *RMR* as shallow torpor, while a reduction of over 90% was considered deep torpor.

The bats exhibited low activity inside the metabolic chambers (e.g., changing position), regardless of the time of day or season. Nonetheless, the highest 20% of the *MR* values from all periods when bats were not torpid were removed to exclude even periods of low activity and the remainder was defined as resting metabolic rate (*RMR*). The reduction in *MR* during a torpid state compared to the *RMR* could then be calculated. Resting energy expenditure (*REE*) was calculated in *kJ h*^*− 1*^*g*^*− 1*^ by

$$\:REE=\:\frac{\:{}_{resting}{\dot{V}}_{O2}\:*\:{\varDelta\:}_{K}{H}_{{O}_{2}}}{1000}$$

with _resting_$\:\stackrel{\prime }{V}$_*O2*_ being the oxygen consumption in *ml h*^*− 1*^*g*^*− 1*^ of RMR including the oxycaloric equivalent $\:{\varDelta\:}_{K}{H}_{{O}_{2}}$of 20.37 *J ml*^*− 1*^*O*_*2*_ (Heldmaier et al. 2013). The torpid energy expenditure (*TEE*) was calculated in the same way for all periods during which bats were in a torpid state.

### Statistics & model fit

Analyses included 64 individuals (female *N* = 26; male *N* = 38), distributed as shown in Table 1. Data were processed and analyzed using CRAN R-4.3.2 (Core Team 2023). For a detailed list of packages used for processing and analyzing see Supplements List 2. The data were tested for normality and homogeneity of variance, relationships and interactions were checked visually. When response variables were normally distributed, one-way ANOVA (followed by a Tukey post hoc analysis) and two-way ANOVA (if checking for 2 variables and > 2 levels) were used. A Kruskal-Wallis rank sum test (followed by a Dunn’s test post hoc analysis, if data contained 2 Variables and > 2 levels) was used for variables that were not normally distributed. Data are presented as mean values ± standard deviation or standard error; *N* represents the number of individuals; *n* is the number of total measurement points included.

Two linear mixed effect model were fitted, separately for both– euthermic and torpid– physiological states, including total of 53,842 observations across the 64 individuals (Bates et al. 2015). The response variable (*RMR* or *TMR*) was transformed using its square root (sqrtMR) to stabilize the variance and normalization of residuals, as well as the skewness of the data. The full model included *T*_*a*_, season, roost, *BM*, sex and diurnal cycle as fixed effects, with individual bat *ID* as a random effect to account for repeated measurements. The diurnal cycle (day: 06:00 to 18:00, night: 18:01 to 05:59) was included to account for physiological differences within the time series data during the active and inactive phases. To obtain unbiased estimates for the effects and for model comparison and selection, we used backward elimination of model components based on significant variables and lowest AIC (Akaike information criterion) / REML (Residual Maximum Likelihood) and Satterthwaite’s method for t-tests, resulting in the exclusion of *BM* and sex from the final model for both, euthermic and torpid states. For both models, the scaled residuals showed a symmetrical distribution around the median and the variance of the random intercepts for *ID* suggests different baseline levels of sqrtMR for different individuals (Supplements: Model).

## Results

### Temperature conditions

Temperature conditions varied between seasons, with a significantly higher mean *T*_*a*_ of 4.83 °C in the wet season across all roost types (*F*_*1,53840*_*= 12576, P < 0.01*). In the buffered cave, *T*_*a*_ was always constant in the daily cycle, but mean *T*_*a*_ was 3.59 °C higher in the wet season (29.7 ± 0.0 °C during the wet season and 26.1 ± 0.1 °C during the dry season; *F*_*1,20339*_*= 803745, P < 0.01*; Table 1). In contrast, the forest habitat exhibited substantial daily temperature fluctuations of 19.1 ± 3.4 °C in the dry season and 8.4 ± 2.0 °C in the wet season (Table 1; Supplements Fig. 1) and mean *T*_*a*_ was 6.43 °C higher during the wet season (*F*_*1,16013*_*= 2808, P < 0.01*). The daily *T*_*a*_ fluctuations in the open cave were similar during both the wet season and dry season (5.65 ± 1.09 °C and 7.61 ± 0.74 °C), but mean *T*_*a*_ was 6.07 °C higher during the wet season (*F*_*1,17484*_*= 25499, P < 0.01;* Table 1).

### Body condition

There were no significant differences in body mass (*F*_*2,65*_*= 0.386, P = 0.682*) or forearm length (*F*_*2,65*_*= 2.741, P = 0.072*) among the roost types. Across both seasons, bats in the forest weighed an average of 9.79 ± 1.15 g, while bats in the open cave weighed 9.55 ± 1.14 g and those in the buffered cave 9.84 ± 1.26 g (Table 1). The only significant difference observed was in BM between males and females (*F*_*2,66*_*= 14.864, P < 0.001*). Across all roost types, males were, on average, 1.32 ± 0.16 g heavier than females. Males weighed an average of 10.27 ± 0.93 g, while females were 8.95 ± 1.01 g. BM was significantly higher at all roost types during the dry season (*F*_*1,66*_*= 9.285, P = 0.003*).

### Body temperature

The average *T*_*sk*_ of euthermic bats differed significantly among roosts (*F*_*2,56*_*= 8.797, P < 0.001*) and between seasons (*F*_*1,56*_*= 6.648, P = 0.013;* Table 1), but not between sexes. The highest euthermic *T*_*sk*_ of 40.5 °C was recorded for a male from the open cave in the morning, while it tried to maintain euthermia just before entering deep torpor. The bats’ average torpid *T*_*sk*_ was significantly lower in the forest (*F*_*2,26*_*= 5.231, P = 0.012*) than in the other two roost types, due to lower *T*_*a*_ while being torpid (Table 1). Almost all individuals in the forest entered deep torpor daily, thereby thermoconforming to *T*_*a*_ for most of the day. The lowest torpid *T*_*sk*_ recorded was 7.9 °C with a *TMR* of 0.33 ml O_2_ h^− 1^ g^− 1^, in an individual during the early morning hours, when *T*_*a*_ was already increasing and at 8.1 °C. Conversely, bats in the buffered cave did not thermoconform most of the time because they either maintained euthermia while within their *TNZ*, or used shallow short torpor bouts (see below: *Torpor patterns*). The highest torpid *T*_*sk*_ recorded was 39.4 °C with a *TMR* of 0.11 ml O_2_ h^− 1^ g^− 1^, from a female in the buffered cave (*T*_*a*_ = 26.6 °C) that expressed short torpor bouts lasting 10 to 30 min (Fig. 2C).

### Metabolic rate & torpor

**Fig. 4:**
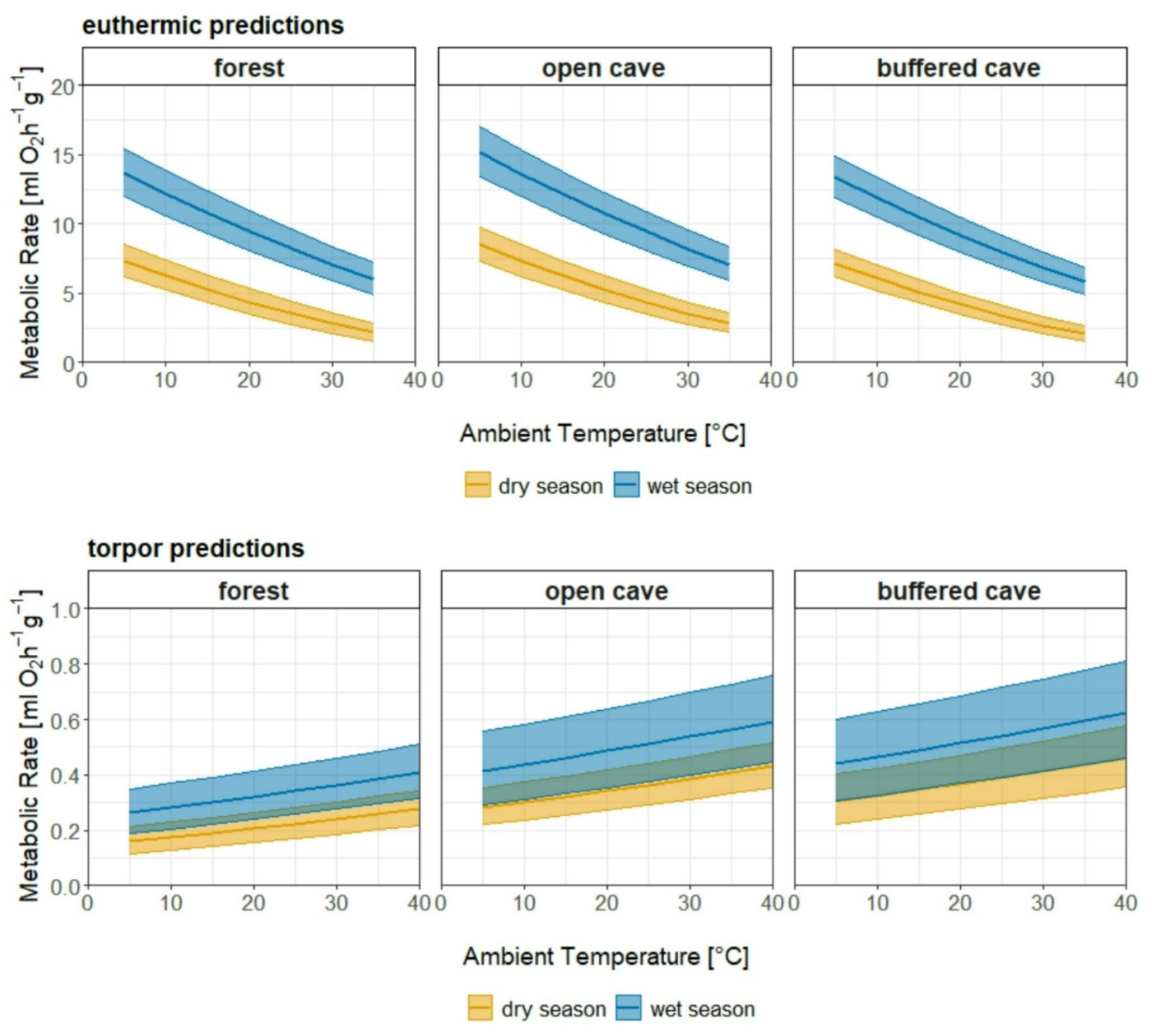
Model predictions for the change of the overall RMR and TMR as a function of T_a_, separately for roosts and seasons. Average MR for every 5 °C interval in T_a_, including the 95% confidence intervals (CI)

Of the 64 individuals, we measured 47 entered torpid states, including deep or shallow torpor (Table 1; Fig. 4). During the dry season (*N = 39*), nearly 90% of the bats (*N = 34*) entered a state of torpor, whereas only 52% (*N = 13*) did so during the wet season (*N = 25*).

There were no significant differences between the sexes in euthermic *RMR* and *TMR*. However, *RMR* and *TMR* differed significantly among the different roosts (*X*^*2*^_*2*_ *= 4108.8, P < 0.01*) and between seasons (*X*^*2*^_*1*_ *= 11505, P < 0.01*). Generally, *MR* was higher during the wet season. During this season, both *RMR* and *TMR* increased along a gradient of decreasing environmental fluctuations in the roosts. The highest *MR* occurred in the buffered cave (Fig. 1).

During the dry season, *TMR* showed the same pattern, while average *RMR* was highest in the forest and lowest in the buffered cave (Fig. 1). Since not all individuals experienced the entire range of *T*_*a*_, we also tested whether *RMR* differed between seasons and roost types only for temperatures that were experienced in all roosts (25 °C to 29 °C). Within this *T*_*a*_ range, the differences in *RMR* between seasons (*X*^*2*^_*1*_ *= 11854, P < 0.01*) and roost types (*X*^*2*^_*2*_ *= 4241.6, P < 0.01*) were also significant, exhibiting the same trend as for the entire temperature range, namely a higher *RMR* during the wet season, with highest *RMR* in the forest and lowest in the buffered cave.

**Table 1:** Mean values (mean), minimum (min), maximum (max) and range (fluc) of ambient temperature (T_a_) and relative humidity (RH_a_) are presented as mean values ± standard error of each measurement day, separately for seasons and roost types. Body mass (BM) and forearm length (FL), resting metabolic rate (RMR), torpid metabolic rate (TMR), euthermic and torpid skin temperature (T_sk_), resting energy expenditure (REE) and torpid energy expenditure (TEE) are presented as mean values ± standard deviation. If sample size differs from the total, it is denoted in parentheses. BM values are given separately for females| males due to the significant differences observed between the sexes

	Unit	Dry season			Wet season		
		Forest	Open cave	Buffered cave	Forest	Open cave	Buffered cave
mean *T*_*a*_	[°C]	20.86 ± 0.09	22.88 ± 0.02	26.29 ± 0.01	27.75 ± 0.05	27.27 ± 0.02	29.54 ± 0.00
min *T*_*a*_		11.05 ± 1.15	20.80 ± 0.77	24.98 ± 0.08	23.65 ± 0.46	25.98 ± 0.46	29.22 ± 0.08
max *T*_*a*_		30.09 ± 2.56	25.84 ± 0.27	27.14 ± 0.12	31.15 ± 1.21	29.78 ± 0.52	30.08 ± 0.18
fluc *T*_*a*_		19.04 ± 3.41	5.04 ± 0.78	2.17 ± 0.61	7.49 ± 1.44	35.81 ± 0.79	0.86 ± 0.18
mean RH_a_	[%]	56.59 ± 0.28	83.63 ± 0.07	81.03 ± 0.04	87.63 ± 0.16	78.01 ± 0.14	89.82 ± 0.07
min RH_a_		30.08 ± 6.21	69.64 ± 1.86	75.88 ± 0.92	76.12 ± 4.24	69.13 ± 3.77	83.83 ± 1.78
max RH_a_		81.67 ± 4.94	92.02 ± 1.85	88.10 ± 1.05	98.04 ± 1.15	93.60 ± 2.79	97.59 ± 1.57
fluc RH_a_		51.58 ± 9.37	22.38 ± 2.82	12.22 ± 0.91	27.47 ± 6.93	24.47 ± 3.04	13.76 ± 1.52
total sample size	*N* (f|m)	11 (5|6)	12 (5|7)	16 (5|11)	8 (6|2)	9 (4|5)	8 (1|7)
BM (*f|m*)	[g]	*f* 10.35 ± 0.84	*f* 7.95 ± 0.37	*f* 9.25 ± 1.32	*f* 8.46 ± 0.25	*f* 9.91 ± 0.92	*f* 8.70 ± 0.88
		*m* 10.63 ± 0.90	*m* 10.21 ± 0.46	*m* 10.95 ± 0.62	*m* 9.88 ± 0.18	*m* 9.92 ± 1.08	*m* 9.31 ± 0.89
FL	[mm]	50.62 ± 1.17	46.88 ± 1.99	47.96 ± 2.37	49.08 ± 1.35	49.96 ± 2.05	49.33 ± 3.42
bats using torpor	*N* [%]	11 (100%)	12 (100%)	11 (68.75%)	8 (100%)	4 (44.44%)	1 (12.5%)
RMR	[ml O_2_ h^− 1^g^− 1^]	7.52 ± 0.90	5.71 ± 0.77	3.09 ± 0.85	7.81 ± 1.34	9.11 ± 1.10	9.22 ± 1.23
TMR		0.27 ± 0.14	0.40 ± 0.17	0.42 ± 0.27 (*N* = 11)	0.39 ± 0.23	0.52 ± 0.28 (*N* = 4)	0.89 ± 0.42 (*N* = 1)
MR reduction	[%]	96.48	93.35	87.56 (*N* = 11)	95.05	94.28 (*N* = 4)	90.61 (*N* = 1)
euthermic T_sk_	[°C]	35.16 ± 1.03	36.30 ± 1.22	36.99 ± 0.83	33.50 ± 1.41	35.30 ± 1.01	36.13 ± 1.23
torpid T_sk_		23.72 ± 5.48	25.86 ± 1.18	33.11 ± 0.86 (*N* = 11)	29.26 ± 3.32	27.84 ± 1.86 (*N* = 4)	34.54 ± 0.59 (*N* = 1)
REE	[kJ h^− 1^ g^− 1^]	0.15 ± 0–03	0.12 ± 0.02	0.07 ± 0.02	0.16 ± 0.06	0.18 ± 0.07	0.19 ± 0.06
TEE		0.005 ± 0.003	0.007 ± 0.005	0.008 ± 0.005 (*N* = 11)	0.008 ± 0.005	0.01 ± 0.005 (*N* = 4)	0.02 ± 0.009 (*N* = 1)

### Torpor patterns

The model we used (see below; Table 2) was designed to analyze factors affecting *MR* in response to different environmental conditions. However, the physiological and thermoregulatory responses revealed intraspecific variation in torpor patterns, with varying levels of *MR* reduction. The variation in torpor patterns were often related to the different microclimates (Fig. 2).

Deep torpor, the most commonly observed response, was characterized by a rapid entry into a torpid state, using only about 5% of the energy compared to a euthermic state (Table 1). This was accompanied by a significant decrease in *T*_*sk*_ until bats thermoconformed with *T*_*a*_, as shown by an individual roosting in the open cave during the wet season (Fig. 2A). Shallow torpor was also frequently observed, with a stable *TMR* around 25% of *RMR* (Fig. 2B). Short torpor bouts were exclusively recorded in bats from the buffered cave, lasting 10 to 30 min and followed by arousal (Fig. 2C). Here, *T*_*sk*_ mirrored *MR*, ranging ~ 8 °C, despite a constant *T*_*a*_ of around 26 °C. Multiple torpor patterns were recorded for some individuals. Bats in the forest displayed shallow torpor bout lasting several hours, reducing *T*_*sk*_ to approximately 20 °C, before entering deep torpor in the morning while thermoconforming to *T*_*a*_, and minimizing *MR* (Fig. 2D).

We focused on how metabolic responses changed with *T*_*a*_ variation. Bats expressed torpor at almost all environmental conditions albeit using various patterns, without a distinct temperature threshold for entry or clear trends of *TMR* as a function of *T*_*a*_ across the roosts and seasons (Fig. 3). In all roosting sites, the bats’ metabolic responses to changes in temperature during euthermia corresponded to expected patterns, with *RMR* decreasing as *T*_*a*_ increased until reaching the presumed *TNZ* (Fig. 3A). In the buffered cave, there was little variation in *T*_*a*_ and *RMR* remained constant, except at high *T*_*a*_, where *RMR* showed a sudden drop at 32 °C. During the dry season, we observed a broader range and a shift of the *TNZ* towards lower *T*_*a*_ across all roost types (Fig. 3A). This trend was particularly evident in the open cave, where *RMR* increased with decreasing *T*_*a*_ during the wet season, plateauing at ~ 29–32 °C. In the dry season, *RMR* plateaued at a lower *MR* between 20 and 25 °C (Fig. 3A).

### Model predictions

To investigate the factors influencing *MR* variability in *T. menamena*, we used Linear Mixed-Effect Models. The significance of the effects of the final models is indicated by the p-values, and the direction and magnitude are indicated by the coefficients (Table 2). Fixed effects analysis for euthermic bats revealed a significant influence of *T*_*a*_, season and the diurnal cycle, while the effects of the roosts were not significant (Table 2). The wet season had the strongest effect on *sqrtRMR*, with an estimated increase of 0.983 ± 0.11 ml O_2_ h^− 1^ g^− 1^ compared to the dry season (*t = 9.050, P < 0.001*). The euthermic and torpor data consistently demonstrated significantly lower *RMR* and *TMR* values during the dry season (Fig. 4). *T*_*a*_ negatively influenced *sqrtRMR* (*t = -42.923, P < 0.001*), indicating an increased *RMR* at lower temperatures (Fig. 4– *euthermic predictions*). During torpor, this was reversed, with *T*_*a*_ having a modest yet statistically significant positive effect on *sqrtTMR* (*t = 10.845, P < 0.001*) (Fig. 4– *torpor predictions*). Furthermore, populations in both open cave and buffered cave were associated with a significantly higher *sqrtTMR* than predicted for the forest bats (Table 2).

**Table 2:** Results of the linear Mixed-Effect models analysis on square root metabolic rate (sqrtMR), separately for the euthermic and torpid physiological State. Showing fixed effects of environmental factors, roost type and diurnal patterns as predictors

euthermic state						
Fixed effects	Estimate	Std. Error	df	t -value	Pr(>|t|)	
(Intercept)	2.920	0.114	65.48	26.208	< 0.001	***
Tambient	-0.041	0.001	36560.00	-42.923	< 0.001	***
season_WS	0.983	0.109	63.30	9.050	< 0.001	***
roost_open cave	0.203	0.140	63.24	1.443	0.154	
roost_buff cave	0.041	0.131	63.62	-0.313	0.756	
cycle_night	0.150	0.003	36560.00	54.623	< 0.001	***
torpid state						
Fixed effects	Estimate	Std. Error	df	t -value	Pr(>|t|)	
(Intercept)	0.383	0.032	36.62	12.059	< 0.001	***
Tambient-	0.004	0.000	16140.00	10.845	< 0.001	***
season_WS	0.112	0.045	31.10	2.480	< 0.018	*
roost_open cave	0.131	0.043	31.92	3.068	0.004	**
roost_buff cave	0.151	0.049	31.10	3.077	< 0.004	**
cycle_night	0.075	0.007	16140.00	11.470	< 0.001	***

The predictions drawn from the models were back-transformed (squared), to show the effects based on the original scale of the response variable. Notably, the predictions from the model did not account for different ranges of *T*_*a*_ specific to each roost, nor did they include a threshold at which individuals transitioned between euthermic and torpid states based on *T*_*a*_. Consequently, we found generalized predictions across the entire temperature spectrum for all habitats and both physiological states, despite the fact that certain roosts may not experience all environmental conditions.

## Discussion

Consistent with our hypotheses, we found differences in *MR* and variation in torpor patterns among *T. menamena* populations, dependent on the type of roost. Populations inhabiting distinct roosts, exhibited physiological adaptability. This finding suggests intraspecific flexibility to different microclimates. Such adaptability may be critical for survival in diverse microclimates, particularly for widespread species in the face of changing environments due to climate change.

### Roost-related variation and seasonal shifts in the thermoneutral zone

Bats in the buffered cave likely roost within their *TNZ* (Reher et al. 2022), gaining additional thermoregulatory advantages from aggregating in larger colonies (e.g. trapping body heat by clustering, reducing EWL and lowering predation risk) (Cardiff 2006; Rodriguez-Duran 1998; Roverud and Chappell 1991). Within the *TNZ*, bats can maintain euthermia at minimal energetic cost, while still engaging in normal physiological and social activities, remaining alert and foraging (Baudinette et al. 2000; Turbill 2006b). Furthermore, high *RH*_*a*_ reduces thermal conductance, resulting in significant conservation of energy and water (McGuire et al. 2021). This assumption is supported by our data demonstrating that bats from the buffered cave exhibited higher *T*_*sk*_ in both euthermic and torpid states, while their *REE* during their active phases was either lower or comparable to that of bats from the other roosts. However, the sample size of *N* = 1 torpid individual during the wet season in this roost is insufficient to compare between seasons.

Our findings suggest that the *TNZ* for *T. menamena* varies among different roosts and shifts with season. This is similar to *M. commersoni*, which roosts in the same habitats but experiences heat stress at different levels (Reher et al. 2022). Seasonal adaptations in thermoregulatory patterns are evident in other (sub-) tropical bats and mammals, such as the blossom bat *Syconycteris australis* or the reddish-grey mouse lemur *Microcebus griseorufus*, which save energy by shifting their *TNZ* to more frequently encountered *T*_*a*_ (Coburn and Geiser 1998; Kobbe et al. 2014). Similar patterns are also observed in small mammals from more temperate climates, such as the striped hamster (*Cricetulus barabensis*), which exhibits a shift in *TNZ* towards lower *T*_*a*_, reducing the critical temperature by approximately 10 °C during winter (Liao et al. 2023). Our study was conducted in natural microclimates, thus not all populations were assessed across the entire range of *T*_*a*_. Consequently, we were unable to identify the *TNZ* for different populations and seasons. However, we observed shifts of the lower and upper critical temperatures between seasons, particularly in habitats with a wider range in *T*_*a*_. This led us to conclude, that *T. menamena* likely exhibits a downward shift of the *TNZ* by approximately 5 to 10 °C during the dry season.

Adjustments in the *TNZ* suggest an additional strategy to minimize energy expenditure while maintaining euthermia (Keicher et al. 2022; Marroquin et al. 2023; Reher, Rabarison, Nowack, et al., 2022). Yet, in the context of climate change, such adaptability has its limits. Prolonged exposure to temperatures beyond the *TNZ* may compel individuals to migrate or seek more energetically favorable roosts. This challenge may be exacerbated in Madagascar, where over 90% of the native habitat has been lost or degraded (Myers et al. 2000), placing endemic species, particularly those specialized to certain microhabitats, among the most endangered globally.

### Torpor and its drivers

Bats exhibited torpor across a range of environmental conditions, with no discernable temperature threshold for initiation or consistent trends in *MR* among different roosts and seasons. Overall, the reduction in *MR* during torpor was about 93% relative to *RMR*, highlighting immense energy conservation. Comparable energy savings during torpor have been found in other tropical bats, such as the Pallas’s long-tongued bat *Glossophaga soricina* or the northern blossom bat *Macroglossus minimus* (Kelm and Von Helversen 2007; Bartels et al. 1998). Moreover, the metabolic reduction further diminishes water use, which may be critical amidst intensifying drought conditions (Roverud and Chappell 1991). However, being torpid has drawbacks, such as an increased predation risk, oxidative damage during arousal periods and reduced responsiveness to social interactions (Choi et al. 1998; Radzicki and Hejduk 1999; Staples and Brown 2008). Consequently, the frequency of torpor use represents a complex tradeoff between various factors.

Our linear mixed-effects models revealed contrasting patterns for *RMR* and *TMR* with changing *T*_*a*_ and varying impacts of environmental factors on *MR*. Substantial variation in *MR*, body temperature and energy expenditure was expected between active and inactive phases across the diurnal cycle (Van Der Vinne et al. 2015). Although bats could not employ normal activity during their active phase due to being held captive in the metabolic chamber, there was a substantial reduction in MR during the day as a result of entering torpor.

Similar to the sub-tropical eastern long-eared bat *Nyctophilus bifax*, that showed an increase in torpor use in warmer relative to the colder seasons(Stawski and Geiser 2010a), *T. menamena* increased use of torpor and especially deeper torpor during the dry season, resulting in a lower *TMR* compared to the wet season. The significantly higher *RMR* during the wet season were evident in all roost types. The seasonal adjustment of *RMR* among small mammals is well-documented (Genoud 2022). Social opportunities are particularly important during the wet season, when mating and reproduction takes place (Wilde et al. 2018; Cumming and Bernard 1997), which affects thermoregulation and torpor use (Mzilikazi and Lovegrove 2002). This seasonal impact of the reproductive cycle on thermoregulation might also apply to *T. menamena*, as pregnant and lactating females were captured during the wet season in all roost types (Fernández-Llamazares et al. 2018; Goodman 2011). Unfortunately, the reproductive biology of this species is not well studied, and more information is needed to draw conclusions about the impact of the reproductive cycle on thermoregulation and torpor use. Furthermore, it should be noted that bats were isolated in the metabolic chamber, whereas they would naturally be in groups of various sizes. The physiological responses of free-ranging bats may differ.

The *MR* reduction of *T. menamena* during torpor was most pronounced in roosts with higher environmental fluctuation. A recent study has shown that bats roosting in more exposed habitats predominantly rely on physiological responses for energy management, whereas those roosting in stable and sheltered environments primarily rely on behavioral strategies (Marroquin et al. 2023). This is analogous to predictions for roost selection from Alston et al. (2022), which suggest that the energetic advantages of maintaining euthermia in warmer and more stable environments may outweigh the energetic costs associated with spending less time in torpor.

### Diverse torpor patterns elucidate intraspecific flexibility

The use of torpor was observed in both seasons and in all roost types. Different torpor patterns were evident by bats using different roost types, which highlights physiological adaptations of *T. menamena* and underscores the importance of studying populations found in different areas. *M. commersoni* has a limited physiological adaptability and an inability to adequately compensate for acute microclimate changes, leaving uncertainty regarding the potential for adaptation among individuals from distinct populations over time.

Shallow torpor was primarily observed in bats roosting in the forest and the open cave, indicating that under fluctuating conditions, it may be energetically efficient to maintain a level of torpor that allows for a rapid return to euthermic *MR*. This strategy could mitigate drawbacks associated with torpor, such as increased predation risk due to reduced responsiveness (Geiser 2013; Radzicki and Hejduk 1999) and the risk of “hyperthermic daily torpor” (Lovegrove et al. 2014), during which the body temperature might increase due to thermoconformity, leading to potential lethality (Speakman and Thomas 2003). Energy conservation might have been further enhanced through passive rewarming in more exposed roosts, as also observed in other tropical animals such as other bats and marsupials (Currie et al. 2015; Geiser and Drury 2003; Lovegrove 1999; Turbill 2006a).

Short torpor bouts were only observed in the buffered cave under stable environmental conditions. It may reflect a trade-off between the costs and benefits of short torpor bouts (Reher et al. 2018). The periodic arousals are presumably inexpensive, given the low thermoregulatory costs in the stable, hot cave environment (Lovegrove 1999). During these shorter torpor bouts, the reduction in bats’ body temperature is not as pronounced as in longer-lasting torpid states. However, the reduction in *MR* and thus energy expenditure was still substantial, so that even short torpor bouts (< 30 min) provide considerable energy savings, particularly for small bats (Geiser and Brigham 2012).

When bats exhibited a combination of shallow and deep torpor within a single day, they initially attempted to maintain higher body temperatures. However, sustaining a higher body temperature of around 20 °C was presumably too costly, prompting them to enter deep torpor. The forest presented greater thermoregulatory challenges, with bats experiencing significant fluctuations in body temperature throughout the day. The lowest individual *T*_*sk*_ recorded was 7.9 °C, with the same individual exhibiting the most substantial daily amplitude *T*_*sk*_ fluctuations of 30.33 °C. All bats in the forest became torpid during respirometry and nearly all entered deep torpor, adopting thermoconformy with *T*_*sk*_ following *T*_*a*_ for most of the day.

In contrast to small bats from temperate or subtropical climates, such as the fishing myotis (*Myotis vivesi*) (Salinas-Ramos et al. 2014) and the insectivorous *Nyctophilus bifax* (Stawski and Geiser 2010a), which typically alternate between prolonged torpor or hibernation in winter and daily torpor in summer, *T. menamena* remains active year-round without hibernating. We assumed that *T. menamena* uses daily torpor to cope with energetic challenges. There might be a yet unknown response to changes in *T*_*a*_ and seasonal resource availability, which could involve the ability to enter prolonged states of torpor. Due to measurement constraints– such as a roughly 24-hour period and the need to release individuals before capturing new ones at dawn and food deprivation during respirometry– we were unable to determine if this species uses prolonged torpor. Although we documented arousals before the measurement period ended (*Supplements: arousal example*), most individuals remained torpid until we entered their roosts to stop the measurements. *T. menamena* may potentially use prolonged torpor during extreme environmental conditions and food scarcity.

## Conclusion

Our research highlights the physiological adaptability of *Triaenops menamena*, whose torpor expression varies seasonally. We found a positive correlation between the reduction in metabolic rate and a gradient of the amplitude of fluctuations in environmental conditions, showcasing how bats can effectively navigate the challenges of dynamic habitats. Our modelling provides a general explanation for the *MR* trends across varying *T*_*a*_.

Our key finding is the observation of diverse torpor patterns, including a potential for the use of prolonged torpor under unfavorable conditions (e.g., heavy rain or low insect availability). This highlights the need to study animals in their respective habitats to capture responses to natural ambient conditions, especially for widespread species like *T. menamena*. The variation in body temperature during torpor suggest that, particularly at higher *T*_*a*_, body temperature alone may not be good proxy of torpor use. Inclusion of additional physiological indicators beyond body temperature, such as metabolic rate or heart rate, would be beneficial (Currie et al. 2014; Reher et al. 2018; Shankar et al. 2023).

## Electronic supplementary material

Below is the link to the electronic supplementary material.

Supplementary Material 1

## Data Availability

The datasets generated and analysed during the current study are available in the DRYAD repository, [will follow].
