# Supplementary material for: Intraspecific variation in metabolic responses to diverse environmental conditions in the Malagasy bat Triaenops menamena
Source: J Comp Physiol B. 2025 Mar 20;195(2):247–62. doi: 10.1007/s00360-025-01608-1 (PMC12069135; doi:10.1007/s00360-025-01608-1)
Supplement: Supplementary file 1 — Supplementary Material 1 [file 360_2025_1608_MOESM1_ESM.docx]

# *Supplementary Information*

# Map:


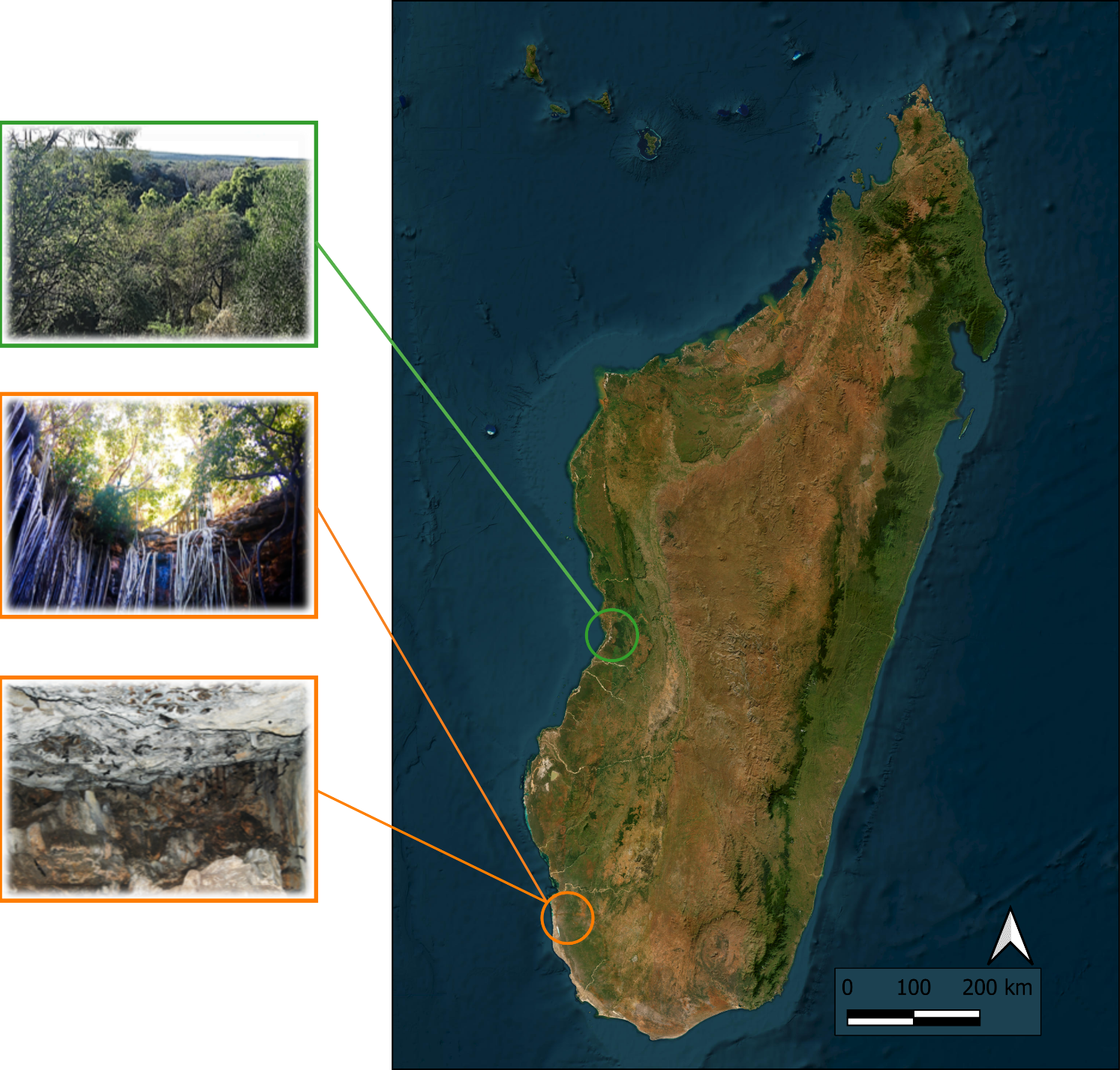
Map of Madagascar with both study areas framed in green (Kirindy forest) and orange (Tsimanampetsotse National Park) in the drier western part of the island. Pictures showing the roosts of the respective study sites.

buffered cave

forest

open cave

# *Environmental Temperature & Humidity*

*Figure 1:* Long-term measurements of temperature and relative humidity during the **wet season** in
***A: buffered cave*** *(Tsimanampetsotse National Park; 2017),* ***B: open cave*** *(Tsimanampetsotse National Park; 2017)*, ***C: forest*** *(Kirindy forest; 2018)*.


*Figure 2:* Long-term measurements of temperature and relative humidity during the **dry season** in
***A: buffered cave*** *(Tsimanampetsotse National Park; 2019),* ***B: open cave*** *(Tsimanampetsotse National Park; 2019)*, ***C: forest*** *(Kirindy forest; 2018)*.

# *Calibration:*

The OxBox operates on a standard 12V car battery and contains electrochemical fuel-cell oxygen sensors (7OX-V CiTicel, Bieler + Lang, Achern, Germany). These O_2_ sensors were calibrated in the laboratory right before and after the field trip. Calibration was achieved using a gas-mixing pump, which generated calibration gases by adding 0%, 3% and 5% N_2_ to the air, respectively (2KM300/a; Wösthoff Messtechnik GmbH, Bochum, Germany). Thereafter, a calibration value corresponding to a 1% reduction in oxygen was calculated using linear regression. The measurement outputs from the OxBox, in [mV], could then be adjusted using the calibration value to determine the O_2_ concentration.

The temperature-sensitive radio transmitters used to record skin temperature were calibrated in advance in a water bath set at temperatures ranging from 3 to 45°C, against a precision thermometer (national standard). The radio signal and pulse period were monitored using a TRX-1000S Receiver and a stopwatch. This monitoring occurred after the transmitters had remained in the water bath for 30 minutes at each of the following temperatures: 3, 10, 17, 24, 31, 38 and 45°C, to ensure equilibration . A least-square regression analysis was employed to determine the relationship between pulse period and temperature, resulting in a regression equation that was subsequently used for calculating skin temperature with an R^2^ greater than 0.99 for all transmitters. The temperature sensitive radio transmitters’ weights were below recommended maxima for bats for load carrying capacity regarding radio telemetry measurements (“5%-rule”; Aldridge & Brigham, 1988).

# *Figure 3:*

Simplified visualization of the experimental setup of the oxygen consumption measurements. The OxBox was connected to the metabolic chamber (red) via gas-tight tubing for the measurement air and the reference air, with interposed pump and silica gel containers. The OxBox is connected to and powered by a 12V battery. During measurements, all components were stored in a metal box to protect the electronical devices from external damages and humidity. (Illustration created with AutoCAD (Autodesk Inc., 2019).

# *List 1: Equipment for field measurements*

- Oxbox: designed and constructed by T. Ruf and T. Paumann, (University of Veterinary Medicine Vienna, Austria).
- temperature sensitive radio transmitter: *Biotrack, Wareham, UK*
- data logger skin temperature: *DataSika SRX-800-D, Biotrack, Wareham, UK*
- iButtons: *Hygrochron iButtons DS1923*
- non toxic ink: *Hauptner-Herberholz, Solingen, Germany*
- local anesthesia cream: *EMLA, AstraZeneca, Wedel, Germany*
- harp trap: *Faunatech Austbat, Bairnsdale, Australia*
- mist nets: *Ecotone, Sopot, Poland*
- medical latex adhesive: *SAUER-Hautkleber, Lobbach; Germany*
- silica gel: *Carl Roth®, Germany*
- gas tight tubing: *Tygon, Saint-Gobain COOP, Courbevoie, France*

# *
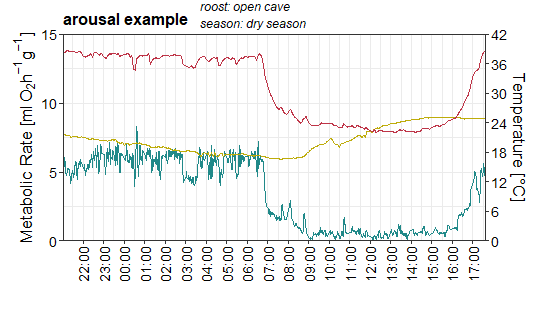
Fig. 4*: *Example of a measurement run of an individual from the open cave during the dry season, including the arousal towards the end of the measurement. Metabolic rate (green), skin temperature(red) and ambient temperature (yellow) over the course of a day.*

*Temperature conditions in the buffered cave*

In the buffered cave, *T. menamena* roosts in large species assemblages with *Macronycteris commersoni* and *Paratriaenops furculus* in the most buffered and flooded chamber of the cave as their preferred roosting chamber (Reher et al., 2019). For this reason, all respirometry measurements were implemented in an adjacent cave chamber, as this avoided unnecessary disturbances of the colony caused by frequent controls of the system, particularly for *M. commersoni* which is hibernating during the colder dry season. Even though the adjoining chamber was marginally colder (mean ΔT_chamber1-2_ = ~3.9°C), the ambient conditions were stable during the whole study (daily temperature fluctuation < 1°C). It is also the main roosting chamber of the species *Miniopterus mahafaliensis,* which is as well active year-round due to similar diet preferences as *T. menamena* (Reher et al., 2019; Russell et al., 2007)

# *List 2: R-packages used for data analyses*

- RStudio (R Core Team, 2023)
- lubridate (Grolemund & Wickham, 2011)
- ggplot2 (Wickham, 2016)
- plyr (Wickham, 2011)
- dplyr (Wickham, François, et al., 2023)
- tidyr (Wickham, Vaughan, et al., 2023)
- tibble (Wickham & Müller, 2023)
- gridExtra (Auguie, 2017)
- RColorBrewer (Neuwirth, 2022)
- colorBlindness (Ou, 2021)
- patchwork (Pedersen, 2024)
- lme4 (Bates et al., 2015)
- nlme (Pinheiro et al., 2023)
- car (Fox & Weisberg, 2019)
- forecast (Hyndman & Khandakar, 2008)
- broom (Robinson et al., 2023)

*References*

Aldridge, H. D. J. N., & Brigham, R. M. (1988). Load Carrying and Maneuverability in an Insectivorous Bat: A Test of the 5% “Rule” of Radio-Telemetry. *Journal of Mammalogy*, *69*(2), 379–382. https://doi.org/10.2307/1381393

Auguie, B. (2017). *gridExtra: Miscellaneous Functions for “Grid” Graphics*. https://CRAN.R-project.org/package=gridExtra

Bates, D., Mächler, M., Bolker, B., & Walker, S. (2015). Fitting Linear Mixed-Effects Models Using **lme4**. *Journal of Statistical Software*, *67*(1). https://doi.org/10.18637/jss.v067.i01

Fox, J., & Weisberg, S. (2019). *An {R} Companion to Applied Regression*. Sage. https://socialsciences.mcmaster.ca/jfox/Books/Companion/.

Grolemund, G., & Wickham, H. (2011). Dates and Times Made Easy with **lubridate**. *Journal of Statistical Software*, *40*(3). https://doi.org/10.18637/jss.v040.i03

Hyndman, R. J., & Khandakar, Y. (2008). Automatic Time Series Forecasting: The **forecast** Package for *R*. *Journal of Statistical Software*, *27*(3). https://doi.org/10.18637/jss.v027.i03

Neuwirth, E. (2022). *RColorBrewer: ColorBrewer Palettes*. https://CRAN.R-project.org/package=RColorBrewer

Ou, J. (2021). *colorBlindness: Safe Color Set for Color Blindness*. https://CRAN.R-project.org/package=colorBlindness

Pedersen, T. L. (2024). *patchwork: The Composer of Plots*.

Pinheiro, J., Bates, D., & R Core Team. (2023). nlme: Linear and Nonlinear Mixed Effects Models. In *Mixed-Effects Models in S and S-PLUS*. Springer-Verlag. https://doi.org/10.1007/b98882

R Core Team. (2023). *R: A Language and Environment for Statistical Computing. R Foundation for Statistical Computing* (Version R 4.3.2) [R]. https://www.R-project.org

Reher, S., Rabarison, H., & Dausmann, K. (2019). Seasonal movements of insectivorous bat species in southwestern Madagascar. *Malagasy Nature*, *13*, 117–124.

Robinson, D., Hayes, A., & Couch, S. (2023). *broom: Convert Statistical Objects into Tidy Tibbles*. https://CRAN.R-project.org/package=broom

Russell, A. L., Ranivo, J., Palkovacs, E. P., Goodman, S. M., & Yoder, A. D. (2007). Working at the interface of phylogenetics and population genetics: A biogeographical analysis of *Triaenops* spp. (Chiroptera: Hipposideridae). *Molecular Ecology*, *16*(4), 839–851. https://doi.org/10.1111/j.1365-294X.2007.03192.x

Wickham, H. (2011). The Split-Apply-Combine Strategy for Data Analysis. *Journal of Statistical Software*, *40*(1). https://doi.org/10.18637/jss.v040.i01

Wickham, H., François, R., Henry, L., Müller, K., & Vaughan, D. (2023). *dplyr: A Grammar of Data Manipulation*. https://dplyr.tidyverse.org.

Wickham, H., & Müller, K. (2023). *tibble: Simple Data Frames.* https://github.com/tidyverse/tibble

Wickham, H. (with Sievert, C.). (2016). *ggplot2: Elegant graphics for data analysis* (Second edition). Springer. https://doi.org/10.1007/978-3-319-24277-4

Wickham, H., Vaughan, D., & Girlich, M. (2023). *tidyr: Tidy Messy Data*.
